# Mannan is a context-dependent shield that modifies virulence in *Nakaseomyces glabratus*

**DOI:** 10.1080/21505594.2025.2491650

**Published:** 2025-04-15

**Authors:** Gabriela Fior Ribeiro, Emily L. Priest, Helen Heaney, Jonathan P. Richardson, Delma S. Childers

**Affiliations:** a Institute of Medical Sciences, Aberdeen Fungal Group, University of Aberdeen, Aberdeen, UK; b Centre for Host-Microbiome Interactions, Faculty of Dentistry, Oral and Craniofacial Sciences, King’s College London, London, UK

**Keywords:** *Candida glabrata*, pathogenesis, cell wall, *MNN10*, *Galleria mellonella*

## Abstract

Fungal-host interaction outcomes are influenced by how the host recognizes fungal cell wall components. Mannan is a major cell wall carbohydrate and can be a glycoshield that blocks the inner cell wall β-1,3-glucan from activating pro-inflammatory immune responses. Disturbing this glycoshield in *Candida albicans* results in enhanced antifungal host responses and reduced fungal virulence. However, deletions affecting mannan synthesis can lead to systemic hypervirulence for *Nakaseomyces glabratus* (formerly *Candida glabrata*) suggesting that proper mannan architecture dampens virulence for this organism. *N. glabratus* is the second leading cause of invasive and superficial candidiasis, but little is known about how the cell wall affects *N. glabratus* pathogenesis. In order to better understand the importance of these species-specific cell wall adaptations in infection, we set out to investigate how the mannan polymerase II complex gene, *MNN10*, contributes to *N. glabratus* cell wall architecture, immune recognition, and virulence in reference strains BG2 and CBS138. *mnn10*Δ cells had thinner inner and outer cell wall layers and elevated mannan, chitin, and β-1,3-glucan exposure compared to wild-type cells. Consistent with these observations, *mnn10*Δ cells activated the β-1,3-glucan receptor in oral epithelial cells (OECs), EphA2, and caused less OEC damage than wild-type. *mnn10*Δ replication was also restricted in macrophages compared to wild-type controls. Yet, during systemic infection in *Galleria mellonella* larvae, *mnn10*Δ cells induced rapid larval melanization and BG2 *mnn10*Δ cells killed larvae significantly faster than wild-type. Thus, our data suggest that mannan plays context-dependent roles in *N. glabratus* pathogenesis, acting as a glycoshield in superficial disease models and modulating virulence during systemic infection.

## Introduction

The fungal cell wall is a complex and dynamic organelle that mediates interactions between fungal cells, the host organism, and the environment. Along with glucans and chitin, mannans make up approximately 30% to 50% of the dry weight of the cell wall – subject to variation pending on fungal genera, species, and growth conditions [1–3]. During mannoprotein synthesis, mannans undergo post-translational modifications through the addition of mannose units to the core protein structure (mannosylation), resulting in the formation of *N*- and *O*-linked mannoproteins [4]. This process occurs within the lumen of the endoplasmic reticulum (ER) and Golgi apparatus and is mediated by a series of proteins (glycosyl and mannosyl transferases, MTases) and enzymatic complexes [4]. The successive steps of mannosylation and structural modifications play a pivotal role in shaping the carbohydrate structure of mannans, significantly impacting their functionality and subcellular localization within the cell wall. The formed *N-*linked mannans typically exhibit longer and more highly branched chains with phosphodiester bonds. In contrast, *O*-linked mannoproteins generally consist of shorter, linear, or unbranched glycans attached via ether linkages to serine or threonine residues of polypeptides [4,5]. The architecture of *N*-mannans is influenced by the fungal species-specific branching and linkage patterns among the composing mannose residues, thereby contributing to the overall diversity of this structure [6]. Regardless of the fungal species, however, the existing paradigm is that *N*-mannans play a crucial role in host–pathogen interactions typically by masking β-1,3-glucan and contributing to immune recognition and/or evasion dependent on different host cell types [6].

The formation of the mannose backbone is contingent on the function of genes within, but not limited to, the Mannan Polymerase II (MPII) complex – namely *ANP1*, *MNN10*, and *MNN11* [7]. These genes encode α-1,6-mannosyltransferases, which are required for mannan backbone extension [7,8]. In *Nakaseomyces glabratus* (formerly *Candida glabrata*), while both *anp1*Δ and *mnn11*Δ yeast cells had shorter mannosyl backbones and increased sensitivity to the cell wall perturbing agents calcofluor white, NaCl_2_, and hygromycin B, each mutant differed in its pathogenic potential [8]. That is, *anp1Δ* cells were hypervirulent in a murine infection model, whereas *mnn11*Δ cells exhibited virulence comparable to the wild-type strain [8]. Cross-complementation between *N. glabratus* and *S. cerevisiae* mutants restored wild-type-like phenotypes, suggesting that *N. glabratus ANP1* and *MNN11* encode functional homologues of the respective *S. cerevisiae* mannosyltransferases [8].

Most of our understanding regarding the role of mannan in *Candida* spp. cell wall composition and host–pathogen interactions stem from *C. albicans* studies. The mannan backbone structures and responses to defects in the initial steps of backbone synthesis appear to be similar between *S. cerevisiae* and *N. glabratus* but diverge from *C. albicans* [7–11]. While both *S. cerevisiae* and *C. albicans* activate a robust cell wall stress response when the earliest steps of backbone synthesis are compromised, *S. cerevisiae* cannot tolerate defects in backbone extension and present severe cell wall and growth phenotypic defects [7]. Specifically, *S. cerevisiae mnn10*Δ and *mnn11*Δ mutant cells display clumped and misshapen morphologies [7]. In contrast, *C. albicans* cells appear to be relatively unaffected by the absence of backbone extension, as the loss of Anp1, Mnn10, or Mnn11 does not yield obvious deleterious effects on cell growth, drug sensitivities, hyphal formation, or macrophage recognition [7]. Importantly, however, there is a *C. albicans* strain-specific effect: changes in cell wall architecture were noted in SN152, but not in BWP17 [7,12]. Deletion of *MNN10* in SN152 alters cell wall polysaccharides, reducing mannan and increasing β-glucan exposure (13). Furthermore, *mnn10*Δ/Δ SN152 cells show significantly attenuated pathogenicity in the murine model despite no effects on macrophage phagocytosis, yeast cell growth, filamentation, or adhesion capabilities (13). Rather, this attenuated pathogenicity is linked to enhanced immune recognition mediated by Dectin-1, not decreased virulence (13). Concurrently, hypo-mannosylation in Anp1/Mnn10-deficient BWP17 cells does not lead to elevated chitin deposition, differing from the compensatory response seen in other yeast and fungi facing cell wall integrity breaches [7]. To illustrate, besides *C*. albicans, this mechanism – where increased chitin content compensates for reduced β-glucan exposure following antifungal treatment – is also observed in *Aspergillus fumigatus* and *A. niger*, for instance [13–15]. Despite strain-specific observations, these findings illustrate that the alternative mechanisms *C. albicans* employs to adapt to weakened cell walls with reduced *N*-linked mannan levels, indicating a distinct mannan tolerance threshold compared to *S. cerevisiae* (7). Further, these findings demonstrate that the role of *N*-linked mannans and associated genes in *C. albicans* may be species-specific due to their considerable differences in chain structure, but also their impact on pathogenesis [6,16–19]. As a consequence, our understanding of how cell wall adaptation and/or remodelling translates into pathogenic outcomes in different model systems is limited. Therefore, characterizing species- and strain-specific mannosylation gene functions is essential to understand how mannan contributes to host–pathogen interactions in diverse niches.

Despite individual MTases playing unique roles within the multimeric complexes, Mnn10 along with Anp1 features as the primary catalyst responsible for forming the majority of the mannan backbone in *S. cerevisiae* [7,20–22]. In *N. glabratus*, *mnn10*Δ cells exhibit reduced survival upon *in vitro* macrophage challenge compared to the wild-type strain [23]. This was linked to a phagolysosome alkalinization defect, implying a potential role for *MNN10* in modulating the environmental pH [23]. Importantly, however, the role of *MNN10* in regulating yeast cell wall features and mediating host–pathogen interactions in *N. glabratus* remains poorly understood. Given the phylogenetic proximity of *N. glabratus* and *S. cerevisiae*, their divergence from *C. albicans*, and variances in *MNN10* function between the latter two species, the present study aimed at addressing the importance of this gene in *N. glabratus* cell wall structure and pathogenesis, both *in vitro* and *in vivo*.

## Methods

### Fungal strains and growth conditions

Wild-type *Candida glabrata* reference strains BG2 and CBS138 (ATCC2001) [24–26] and mutants were maintained by sub cultivation on 2% glucose, 2% bacto-peptone, 1% yeast extract, and 2% bacto-agar (YPD) plates at 37°C from a frozen stock (−80°C). All strains utilized/generated in this study are listed in Supplemental Table S1, with wild-types kindly provided by the Aberdeen Fungal Group. Prior to the experiments, yeast cells were conditioned overnight in 5 mL (37°C, 200 rpm) MOPS-buffered (Morpholino Propanesulfonic Acid) (Melford, Ipswich Suffolk, UK) liquid RPMI-1640 medium (2% glucose, pH 7) (Sigma, MA, USA).

### MNN10 gene deletion in N. glabratus strains

Gene deletion of *MNN10* in both BG2 and CBS138 strains was achieved using the Cre-Lox (Clox) method [27]. Disruption cassettes, described in Supplemental Table S2, were amplified via LongAmp PCR with the following cycling conditions: denaturation at 94°C for 5 minutes, followed by 35 cycles of annealing (1 minute at 94°C, 1 minute at 54°C, 5 minutes at 68°C), and a final extension at 68°C for 10 minutes. The disruption cassettes were transformed into *N. glabratus* yeast cells using the lithium acetate (LiOAc) method [28]. After an overnight incubation at 30°C, 200 rpm, in YPD, followed by further incubation for 4 hours, the cells were transformed when OD600 reached between 0.8 and 1.2. Transformations included centrifugation, washing, and resuspension in a 0.1 M LiOAc solution. After a 15-minute incubation at 30°C, transformation mixtures containing herring sperm ssDNA, DNA cassette, 50% PEG3350, and 1 M LiOAc were incubated for 30 minutes at 30°C, followed by addition of DMSO and a 42°C heat shock for 20 minutes. Post-transformation, cells were incubated overnight, and confirmation was performed by PCR of colonies grown on selective plates containing 100 μg/mL nourseothricin (NOU) (Jena Biosciences, Thuringia, Germany). Cassette flip of deletion mutants (Clox cassette) involved incubating cells in 10 mL YPD overnight at 30°C, 200 rpm. The next day, the cells were spread onto YPD and YPD containing 20 µg/mL NOU. After 48 hours at 30°C, NOU-sensitive colonies were picked and re-streaked for confirmation on YPD and/or YPD with 100 µg/mL NOU. Colony PCR with Dreamtaq cycling conditions (95°C for 2 minutes, followed by 40 cycles of 95°C for 30 seconds, 52°C for 30 seconds, and 72°C for 1 minute 15 seconds) confirmed both deletion and addback using primers in Supplemental Table S2.

### pCN-PDC1 plasmid cloning

*MNN10* gene cloning into the pCN-PDC1 plasmid utilized SpeI and XhoI (New England Biolabs, MA, USA) restriction enzymes, with ORF sequences detailed in Supplemental Table S2. The plasmid was digested, and *MNN10* sequence was inserted using these enzymes, and ligations transformed into Zymo Mix ‘n Go DH5α *Escherichia coli*. pCN-PDC1 plasmids containing *MNN10*, or empty vectors were transformed into wild-type and *mnn10*Δ strains in BG2 and CBS138 using the LiOAc method.

### Fluorescence staining and flow cytometry

Fungal cells were grown overnight in RPMI-1640, followed by overnight inactivation in 50 mm thimerosal (Sigma, MA, USA). Cells were then washed three times with PBS and counted by haemocytometer. To analyse cell wall carbohydrate exposure, 2.5 × 10^6^ cells were stained with Fc-Dectin-1 (provided by Gordon Brown, MRC-CMM), and goat anti-human IgG antibody conjugated to Alexa Fluor 488 (Invitrogen, MA, USA), Wheat Germ Agglutinin (WGA) conjugated to Alexa Fluor 680 (Invitrogen, MA, USA), and Concanavalin A (ConA) conjugated to Texas Red (Invitrogen, MA, USA). Data were acquired for a minimum of 20,000 events on an Attune NxT (Thermo Fisher) and analysed using FlowJo v10 software (TreeStar Inc., OR, USA). Gating strategy is provided in Supplemental Figure S1.

### Transmission electron microscopy (TEM)

Yeast cells, grown overnight in 5 mL RPMI at 37°C and 200 rpm, were back-diluted to 1 × 10^8^ cells and further cultured for 4 hours in 100 mL RPMI. Sample processing as previously described [29]. After centrifugation, the pellet was frozen using a Leica EMPACT2 high-pressure freezer (Leica Microsystems, Wetzlar, Germany). Freeze substitution followed the program in Supplemental Table S3, with subsequent embedding in Spurr’s resin. Sections of 90 nm were prepared and viewed on a JEM 1400 plus Transmission Electron Microscope (JEOL, Tokyo, Japan). ImageJ (Fiji) was used to measure the thickness of the inner (chitin and glucan) and outer (mannan) cell walls. Microscopy was conducted in the University of Aberdeen’s Microscopy and Histology Core Facility.

### Ethical statement

Work involving mouse tissues was approved by the University of Aberdeen Animal Welfare and Ethical Review Body and complied with all ethical regulations. Mice did not undergo any procedures prior to culling with cervical dislocation and subsequent femur removal.

### Culture of murine bone marrow-derived macrophages (BMDMs) and yeast challenge

Bone Marrow-Derived Macrophages (BMDMs) were isolated from 12-week-old C57BL/6 mice [30,31], which were a kind gift from Gordon Brown and selected from specific-pathogen-free in-house breeding colonies at the University of Aberdeen, in accordance with ARRIVE guidelines. BMDMs were cultured in DMEM supplemented with 10% Fetal Calf Serum, 15% L929 cell conditioned medium, 1% L-glutamine, and 1% Penicillin/Streptomycin. For macrophage interaction studies, 3 × 10^4^ BMDMs were plated and incubated overnight at 37°C, 5% CO_2_. Yeast cells, grown overnight in RPMI-1640, were added to BMDMs at a 3:1 MOI. Two hours post-challenge, the supernatant was replaced, and unengulfed yeast cells were removed. For the 2-hour timepoint, 100 µL of 0.02% chilled SDS (Sodium Dodecyl Sulphate) (Melford, Ipswich Suffolk, UK) was added to each well, its contents were scraped, and cell lysates were plated to determine CFU/mL. The same procedure was repeated after 24 hours of co-culture.

### *Galleria mellonella* infection, survival, and melanization

*G*. *mellonella* larvae were purchased from Livefood UK Ltd. (Axbridge, UK) and stored in wood shavings in the dark at room temperature prior to experimentation. Specific details of each experiment are detailed in the appropriate figure legends. Briefly, *N. glabratus* yeast cells (wild-types and/or mutants) were grown overnight in 6 mL RPMI-1640 medium, at 37°C, 200 rpm. Cells were then washed and resuspended in sterile PBS. Larvae (~250 mg weight) were randomly allocated into groups (specific sample sizes indicated on figure legends) and infected in the last left proleg with 5 × 10^6^ yeast cell suspension in 50 µL 1× PBS/larvae using a U-100 30 G Micro-fine syringe (BD, NJ, USA). Control groups were injected with 50 µL sterile saline only. Larvae were incubated at 37°C in the dark, and survival and melanization were assessed daily for a period of 144 hours. Scoring of larval melanization was performed as described previously [32]. Briefly, larvae were considered partially melanized when their natural colour changed but their bodies were not yet fully darkened; they were considered fully melanized when their colour completely changed to dark grey or brown. Dead larvae were also counted as fully melanized [32]. Melanin production was also quantitatively assessed as described previously [33], with minor modifications. Groups of 20 larvae were inoculated with *N. glabratus* as described above, with a control group of eight larvae inoculated with 50 µL PBS, and incubated for 2 h before decapitation with sterile scissors. Twenty µL of haemolymph were collected per larvae and transferred to a well in a clear, flat-bottomed 96-well plate containing 100 µL anti-coagulant solution (93 mm NaCl, 100 mm glucose, 30 mm trisodium citrate, 26 mm citric acid, and 10 mm Na_2_EDTA, pH 4.6, and filter sterilized with a 0.2 µm filter) and placed on ice. Sample absorbance was measured at 405 nm on a VersaMax microplate reader using Softmax Pro software (Molecular Devices, San Jose, CA, USA), with an equivalent volume of anti-coagulant buffer used as a blank. The absorbance values for PBS-injected larval haemolymph were averaged, and that average background reading was subtracted from all *N. glabratus*-injected larval haemolymph absorbances to normalize spectra readouts.

### Mammalian TR146 cell culture

All experiments were performed using the TR146 human oral epithelial cell line [34] purchased from the European Collection of Authenticated Cell Cultures. Cultures were verified to be mycoplasma-free by PCR. Cells were cultured in DMEM – F-12 nutrient mixture (1:1) plus L-glutamine (Gibco, MA, USA) supplemented with 15% (vol/vol) heat-inactivated foetal bovine serum and 1% (vol/vol) penicillin–streptomycin (Thermo Fisher Scientific, MA, USA) at 37°C and 5% CO_2_. Cells were cultured until confluent, and DMEM-F-12 was replaced with serum-free medium for 24 hours.

### Infection of TR146 cells

*Candida* sp. yeast cells were cultured on YPD solid medium (1% yeast extract, 2% peptone, 2% glucose, 2% agar) at room temperature. Single colonies were inoculated into 5 mL of YPD liquid medium and cultured overnight at 30°C in a shaking incubator at 180 rpm. Next day, fungi were washed twice in sterile PBS, and cell density was determined by measuring absorbance (OD_600_). For western blotting, epithelial cells were infected with fungi at a multiplicity of infection (MOI) of 10 for 2 hours. For cytokine assays, epithelial cells were infected at an MOI of 1 (*N. glabratus*) or 0.01 (*C. albicans*) and incubated at 37°C, 5% CO_2_ in a humidified incubator for 24 hours.

### Epithelial cell damage assay

Damage to epithelial cells was quantified using a CytoTox 96 Non-Radioactive Cytotoxicity Assay Kit (Promega, WI, USA) according to the manufacturer’s instructions. Recombinant porcine lactate dehydrogenase (Sigma, MA, USA) was used to create a standard curve.

### Protein extraction from epithelial cells

Tissue culture plates were placed on ice, and cells were washed twice with 1 mL of ice-cold PBS, then lysed with 100 µL of a modified RIPA buffer (25 mm Tris-HCl, pH 7.4, 150 mm NaCl, 1% Nonidet *p*-40 (NP-40), 1 mm EDTA, and 5% glycerol), and supplemented with 1× protease inhibitors and 1× phosphatase inhibitors (Sigma Aldrich, MA, USA). After 2 minutes, a sterile cell scraper was used to detach cells from the surface of the plate. After scraping, crude cell extracts were collected, transferred to microfuge tubes, and incubated on ice for 30 minutes. Following incubation, the extracts were clarified by centrifugation in a benchtop microfuge at 13,300 × *g* at 4°C for 10 minutes. Clarified extracts were collected and protein concentration estimated using a bicinchoninic acid assay (Thermo Fisher Scientific, MA, USA) according to the manufacturer’s instructions.

### SDS-PAGE and western blotting

Proteins were resolved by electrophoresis on Tris-Glycine 4–20% gels (Thermo Fisher Scientific, MA, USA) at 90 V for 3 hours. Electrophoresed proteins were transferred to a nitrocellulose membrane (Bio-Rad, CA, USA) using a Trans-Blot Turbo Transfer System (Bio-Rad). Membranes were washed in 1× Tris-buffered saline (TBS; Severn Biotech) containing 0.1% Tween 20 (Acros Organics, Antwerp, Belgium) and then blocked in TBS-tween containing 5% fat-free milk powder (Sainsbury’s) for 1 hour with gentle shaking at room temperature. EphA2 and p-EphA2 antibodies were diluted (1:1000) in TBS-tween, and 5% milk and membranes were incubated in antibody solution overnight at 4°C with gentle shaking. Following incubation, membranes were washed with TBS-tween (3 × 10 minutes), followed by incubation with a species-specific secondary antibody diluted (1:20000) in TBS-tween for 1 hour at room temperature. Membranes were washed again with TBS-tween (3 × 10 minutes). Proteins were detected using the Immobilon Western Chemiluminescent HRP Substrate (Merck Millipore) and visualized with an Odyssey® Fc Imaging System (LI-COR, NE, USA). Human α-actin was used as a loading control. Percentage of EphA2 phosphorylation was calculated as [total EphA2 versus actin]/[phosphorylated EphA2 versus actin]*100.

### Antibodies for assessment of EphA2 phosphorylation

Anti-EphA2 (D4A2) and anti-p-EphA2 (S897) rabbit monoclonal antibodies were purchased from Cell Signaling Technologies (CST Inc., MA, USA) (catalogue numbers 6997S and 6347S, respectively). Anti-α-actin (clone C4) mouse monoclonal antibody was purchased from Merck (NJ, USA) (catalogue number MAB1501). Peroxidase-conjugated AffiniPure goat anti-mouse and anti-rabbit IgG secondary antibodies were purchased from Jackson Immune Research (PA, USA) (catalogue numbers 115-035-062 and 111-035-003, respectively).

### Quantification of cytokine release from epithelial cells

Exhausted cell culture medium was collected and clarified by centrifugation (200 × *g* at 4°C for 10 minutes). The concentration of cytokines was determined using magnetic microparticles (R&D Systems, MN, USA) specific for human IL-1α, IL-1β, IL-6, G-CSF, and GM-CSF using a Magnetic Luminex Performance Assay (Bio-Techne) and Bio-Plex 200 System (Bio-Rad, CA, USA) according to the manufacturer’s instructions. Data were analysed using Bioplex Manager 6.1 software (Bio-Rad, CA, USA).

### Statistical analyses

Statistical analyses were performed using GraphPad Prism v10.2.3 software (GraphPad Software, CA, USA), and IBM SPSS Statistics v27.0 (IBM Corp., NY, USA). Specific experimental analyses are described in figure legends. Power analyses were used to estimate sample sizes required to achieve statistically robust differences and were based on data generated in previous experiments. Macrophage-yeast survival was analysed by Kruskal−Wallis with Dunn’s multiple comparisons test. Flow cytometry was analysed by One-Way ANOVA with Tukey’s multiple comparisons test. TEM measurements were analysed by Two-Way ANOVA with Tukey’s multiple comparisons test. The *G. mellonella* survival was analysed by Kaplan−Meier, Log-Rank pairwise over strata. Haemolymph absorbance within each strain background was analysed by Mann-Whitney U test. Pro-inflammatory cytokine release and cell damage (LDH, lactate dehydrogenase) were analysed by Grubbs Test with alpha set at 0.05 to identify and remove outliers and then One-Way ANOVA with Tukey’s multiple comparisons test. A *p* value of <0.05 was considered to be significant, and the results are shown as mean ± standard deviation (SD).

## Results

### MNN10 plays a role in maintaining the N. glabratus cell wall

**Figure 1.**
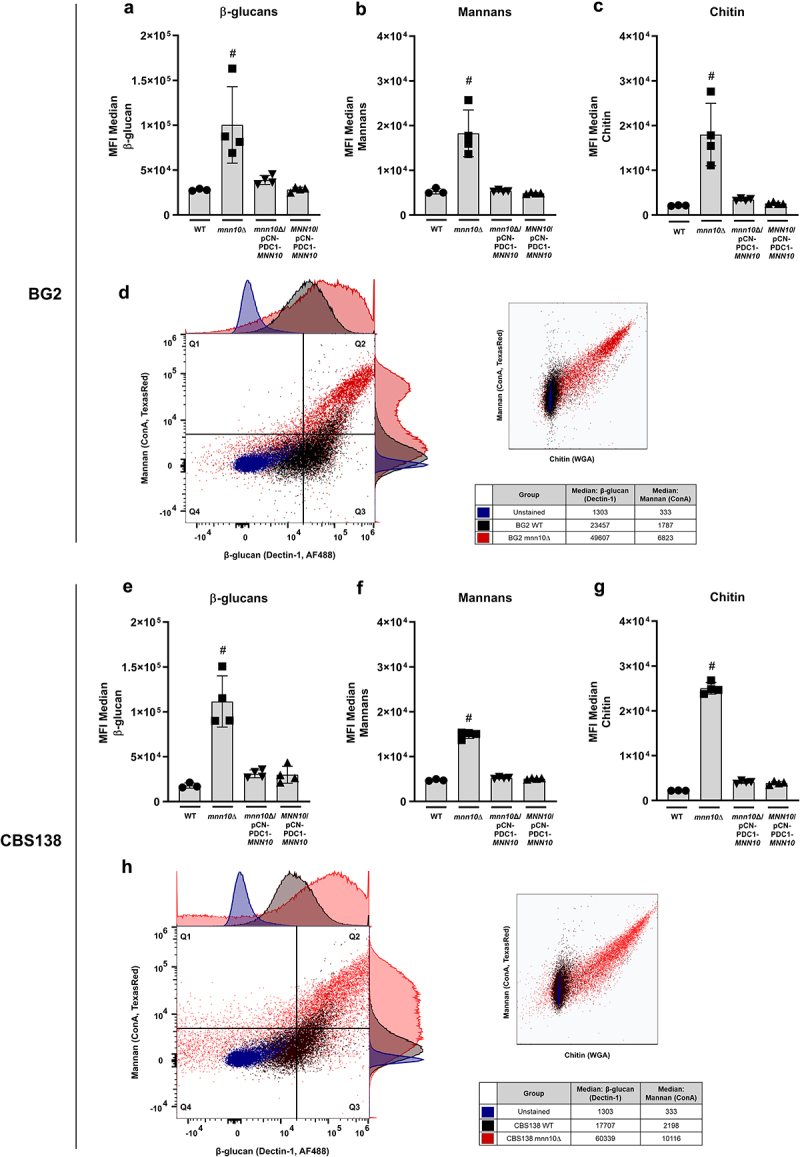
Deletion of MNN10 leads to increased exposure of cell wall polysaccharides in both BG2 and CBS138 N. glabratus backgrounds. Median fluorescence intensity (MFI) comparison of BG2 WT, mnn10Δ, and MNN10/pCN-PDC1-MNN10 for β-glucan (a), mannan (b) and chitin exposure (c). (d) Quartile MFI comparison of BG2 WT and mnn10Δ for β-glucan and mannan, and mannan and chitin. MFI comparison for CBS138 WT, mnn10Δ, and MNN10/pCN-PDC1-MNN10 of β-glucan (e), mannan (f) and chitin exposure (g). (h) Quartile MFI comparison of CBS138 WT and mnn10Δ for β-glucan and mannan, and mannan and chitin. MFI data represents the median of 3–4 biological replicates/group plotted as mean ± standard deviation (SD). WT, wild-type strain. mnn10Δ, null mutant strain. mnn10Δ/pCN-PDC1-MNN10, ectopic expression (not stably integrated) of the MNN10 gene in the null mutant strain. MNN10/pCN-PDC1-MNN10, ectopic expression (not stably integrated) of the MNN10 gene in the wild-type strain. Fc-dectin-1, β-glucans. Concanavalin a (ConA), mannans. Wheat germ agglutinin (WGA), chitin. ^#^*p* ≤ 0.05 against all remaining groups. Statistical analysis was performed using One-Way ANOVA with Tukey’s multiple comparisons test.

We first wanted to determine how *MNN10* affects cell wall polysaccharide exposure in the two *N. glabratus* reference strain backgrounds, BG2 and CBS138. We observed by flow cytometry that *MNN10* deletion more than doubled the exposure of all cell wall components compared to wild-type cells (i.e. β-glucans, mannans, and chitin detected by Fc-Dectin-1, ConA, and WGA, respectively) (Figure 1(a–g)). The dot plot representation of fluorescence between wild-type and *mnn10*Δ strains in both BG2 and CBS138 backgrounds further shows the increase in exposure of β-glucans and mannans in the absence of *MNN10* (Figures 1(d,h)). In the BG2 background, while the wild-type strain events (black) clustered mostly on Q3 (β-glucan^high^/Mannan^low^), the mutant strain (red) clustered on Q2 (β-glucan^high^/Mannan^high^) (Figure 1(d)). We observed a fold change of 2.11 for β-glucan and 3.8 for mannan exposure for *mnn10*Δ cells compared to the wild-type (Figure 1(d)). In parallel, for CBS138, wild-type events are clustered on Q1/Q2 (β-glucan^low/high^/Mannan^low^) while mutant events are clustered mostly on Q2 (β-glucan^high^/Mannan^high^) (Figure 1(h)). This resulted in a 3.4-fold change in β-glucan and a 4.6-fold change in mannan exposure for *mnn10*Δ cells compared to wild-type (Figure 1(d)).

Complementing *mnn10*Δ strains with an episomal copy of *MNN10* reduced carbohydrate exposure back to wild-type-levels in both strain backgrounds (Figures 1(a–h)). We further assessed whether altering gene dosage for *MNN10* perturbed cell wall carbohydrate exposure. However, cells carrying an additional copy of the *MNN10* gene (in the pCN-PDC1 plasmid) also showed wild-type-like levels of exposure of all cell wall components in both BG2 and CBS138 backgrounds (Figures 1(a–c),(e–g)).

**Figure 2.**
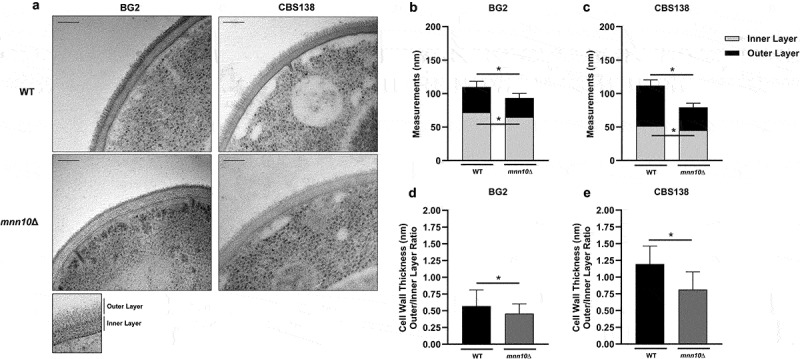
BG2 and CBS138 *N. glabratus mnn10*Δ mutants have reduced inner and outer cell wall layer thickness compared to their respective wild-type controls. (a) Transmission electron microscopy (TEM) comparison of BG2 and CBS138 WT and *mnn10*Δ cell walls. (b) TEM measurements of inner and outer cell wall thickness for BG2 WT and *mnn10*Δ strains. (c) TEM measurements of inner and outer cell wall thickness for CBS138 WT and *mnn10*Δ. (d) BG2 outer/inner cell wall layer thickness ratio. (e) CBS138 outer/inner cell wall layer thickness ratio. Scale bars represent 100 nm. *n* = 20–30 cells/group, 10 measurements/cell (maximum of 256 values plotted). WT, wild-type strain. *mnn10*Δ, null mutant strain. Data plotted as mean ± standard deviation (SD). *p ≤0.05 between indicated groups. Statistical analysis was performed using Two-Way ANOVA with Tukey’s multiple comparisons test.

We also investigated how *MNN10* influences the general appearance of cell wall architecture by Transmission Electron Microscopy (TEM). TEM images showed that, in both BG2 and CBS138 backgrounds, *mnn10*Δ cells had reduced thickness of the outer and inner cell wall layers (Figures 2(a–c)). The inner layer thickness was an average of ~65 nm for *mnn10*Δ versus ~72 nm for wild-type cells, with an outer layer thickness of ~29 nm for *mnn10*Δ versus ~38 nm for wild-type BG2 (Figures 2(a,b)). Similarly, for CBS138, we observed an inner layer thickness average of ~45 nm for *mnn10*Δ compared to ~52 nm for wild-type cells, and an outer layer thickness of ~34 nm for *mnn10*Δ versus ~60 nm for wild-type (Figures 2(a,c)).

We also calculated whether the observed changes in cell wall layer thickness altered the relative proportions of the inner cell wall to the outer layer. The mean ratio of outer to inner cell wall thickness was 0.57 for wild-type and 0.46 for *mnn10*Δ cells in the BG2 background (Figure 2(d)). CBS138 had a more pronounced change in the cell wall thickness ratio with wild-type cells averaging 1.2 compared to 0.81 for *mnn10*Δ cells (Figure 2(e)). These results indicate that the mutant strains, in both backgrounds, presented overall reduced cell wall thickness compared to their respective wild-type controls.

In summary, our data suggest that *MNN10* is required for normal presentation of cell wall polysaccharides in both BG2 and CBS138 *N. glabratus* backgrounds, which might influence host recognition and responses.

### mnn10Δ cells enhance activation of EphA2 in oral epithelial cells

The altered cell wall features observed between wild-type and *mnn10*Δ strains led us to consider what role *MNN10* might have in yeast cell recognition and virulence. First, we investigated the consequences of these changes in a model of superficial candidiasis, utilizing oral epithelial cells (OECs). In OECs, EphA2 is a receptor that participates in β-glucan recognition, triggering the production of pro-inflammatory mediators in response to fungal infections [35]. In neutrophils, EphA2 augments Fcγ receptor-mediated antifungal activity and controls early fungal proliferation (*C. albicans*) during oropharyngeal candidiasis (OPC) [36].

**Figure 3.**
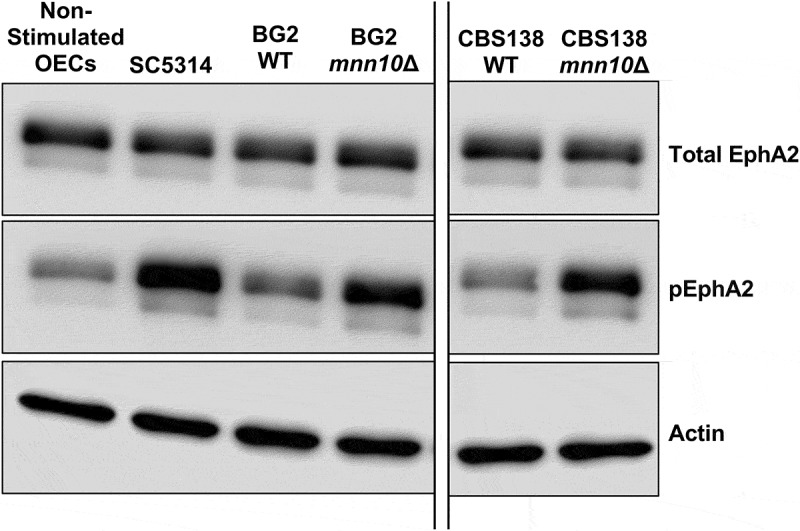
*N. glabratus mnn10Δ* mutants induce higher phosphorylation of OEC EphA2 compared to their respective wild-type parental strains. Western blotting for total and phosphorylated EphA2 (pEpha2). Human α-actin, loading control. Figure is representative of three independent experiments. OECs, oral epithelial cells, negative control. SC5314, *C. albicans* reference strain, positive control. WT, wild-type strain. *mnn10*Δ, null mutant strain.

We used Western blot (WB) analysis to determine EphA2 activation states after OEC challenge with our *N. glabratus* strains with *C. albicans* as a positive control. Total EphA2 receptor expression levels were similar between *N. glabratus* wild-type and mutant strains, *C. albicans*, and unchallenged OECs (Figure 3). As expected, *C. albicans* (SC5314) induced strong phosphorylation of EphA2 (Figure 3). Strikingly, the OECs challenged with *N. glabratus mnn10*Δ from both BG2 and CBS138 backgrounds also exhibited EphA2 phosphorylation (Figure 3). Densitometry analysis indicated that the EphA2 phosphorylation percentage showed an approximately 2-fold increase for BG2 *mnn10*Δ compared to its wild-type and 3.6-fold increase for CBS138 *mnn10*Δ (Table 1).

**Table 1.** Semi-quantitative analysis of EphA2 phosphorylation following OEC challenge with *mnn10*Δ and wild-type *N. glabratus* strains.

	%phospho A	%phospho B	%phospho C	%phospho Mean	Standard Deviation
Non-stimulated OECs	5.2987	4.9324	17.04797	9.0930	6.8916
SC5314	80.4597	24.1054	76.4423	60.3358	31.4407
BG2 WT	18.7833	3.9479	25.1941	15.9751	10.8979
BG2 *mnn10*Δ	31.2661	8.9775	47.0085	29.0840	19.1092
CBS138 WT	16.5202	8.4317	19	14.6506	5.5266
CBS138 *mnn10*Δ	72.1893	29.9465	55.555	52.5636	21.2797
Fold change %phospho (*mnn10*Δ vs WT)					
BG2	1.6646	2.2740	1.8659	1.9348	0.3105
CBS138	4.3698	3.5517	2.9239	3.6151	0.7250

Mean values of western blot pixel measurements from three independent experiments (labelled A-C). OECs, oral epithelial cells, negative control. SC5314, *C. albicans* reference strain, positive control. WT, wild-type strain. mnn10Δ, null mutant strain.

Overall, our data suggests that *N. glabratus MNN10* contributes to maintaining an immune evasive cell wall architecture that limits activation of EphA2 in OECs (Figure 3, Table 1). These data suggest that OECs have increased recognition of β-glucans in *mnn10*Δ cells, which is consistent with our flow cytometry and TEM findings where we observed increased exposure of cell wall polysaccharides, including β-1,3-glucans, as well as reduced outer layer thickness in both *N. glabratus* strain backgrounds (Figures 1 and 2).

### mnn10Δ cells induce GM-CSF release by oral epithelial cells but cause limited host cell damage

**Figure 4.**
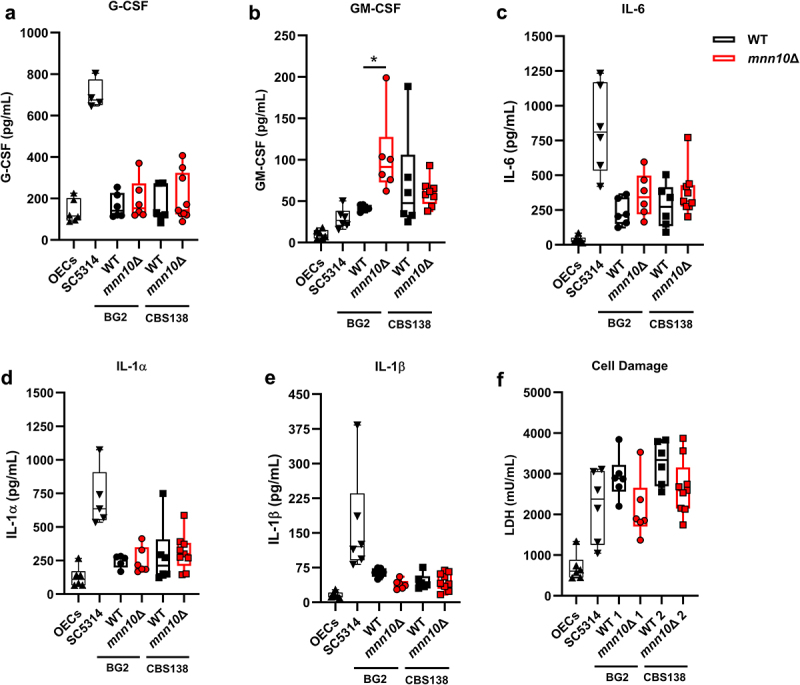
BG2 and CBS138 *mnn10*Δ cells enhance the release of the pro-inflammatory cytokine GM-CSF by oral epithelial cells, but reduce host cell damage following *N. glabratus* challenge. OECs were challenged with *N. glabratus* yeast cells and at 24 hours post-challenge production of GM-CSF (a), G-CSF (b), IL-6 (c), IL-1α (d), and IL-1β (e) was measured. Box and whiskers plot represent minimum to maximum values, showing all points of the dataset. Width distribution of points proportionate to the number of points at that Y value. The whiskers go down to the smallest value and up to the largest; the line in the middle of the box is plotted at the median. *n* = 5–9. (f) Oral epithelial cell damage based on LDH levels. *n* = 5–9. OECs, non-stimulated oral epithelial cells, negative control. SC5314, *C. albicans* reference strain, positive control. WT, wild-type strain. *mnn10*Δ, null mutant strain. **p ≤0.001 between indicated groups. Statistical analysis was performed using one-way ANOVA with Tukey’s multiple comparisons test.

Because EphA2-β-glucan binding primes host cells for an inflammatory response, we next assessed whether the enhanced phosphorylation of EphA2 in response to *mnn10*Δ challenge induced the release of pro-inflammatory cytokines from OECs. We measured the production of the cytokines G-CSF, GM-CSF, IL-6, IL-1α, and IL-1β, which are involved in antifungal host responses (Figures 4(a–e)) [37–42].

We observed that *mnn10*Δ cells in the BG2 *N. glabratus* background (*p* = 0.001) elicited higher GM-CSF production by OECs compared to their respective control (Figure 4(b)). More specifically, the OECs challenged with BG2 *mnn10*Δ yeast cells increased their release of GM-CSF by an average of ~2-fold compared to the wild-type challenge (Figure 4(b)). For CBS138 *mnn10*Δ challenge, however, the release of this cytokine was only increased by ~1.3-fold versus wild-type (Figure 4(b)). Additionally, there was a trend of increased IL-6 and IL-1α release following OECs challenge with *mnn10*Δ *N. glabratus* yeast cells, depending on the strain background (Figure 4(c,d)). On the other hand, we observed no major differences in G-CSF release between wild-type and mutant strains (Figure 4(a)). It is worth noting, however, that for G-CSF, all *N. glabratus* strains elicited similar cytokine levels to the non-stimulated OECs (negative control) (Figure 4(a)), and for GM-CSF all *N. glabratus* strains elicited a stronger immune response (i.e. higher pro-inflammatory cytokine release) than the *C. albicans* positive control (Figure 4(b)). Finally, the challenge of OECs with BG2 *mnn10*Δ yeast cells led to a reduced release of IL-1β compared to its respective wild-type control (Figure 4(e)). In contrast, no clear differences were observed in IL-1β production by OECs challenged with CBS138 *mnn10*Δ and wild-type cells (Figure 4(e)).

Overall, our findings suggest that *MNN10* plays a role in both *N. glabratus* reference strain backgrounds in modulating β-glucan exposure to prevent and/or reduce the release of specific pro-inflammatory cytokines by OECs (Figures 4(a–e)). We initially hypothesized that this increased cytokine elicitation would translate to greater host damage. Contrary to expectations, however, our results indicate that host cells challenged with *mnn10*Δ yeast strains led to reduced oral epithelial cell damage (Figure 4(f)). For both BG2 and CBS138 backgrounds, *mnn10*Δ yeast cells led to a slight reduction of LDH levels by ~0.7- and ~0.8-fold, respectively, compared to their corresponding wild-type controls (Figure 4(f)). Interestingly, although *C. albicans* caused cell damage as expected, *N. glabratus* wild-type strains caused similar (BG2) or higher (CBS138) levels of cell damage than SC5314 (Figure 4(f)).

### Altering MNN10 copy number affects yeast replication in murine BMDMs

**Figure 5.**
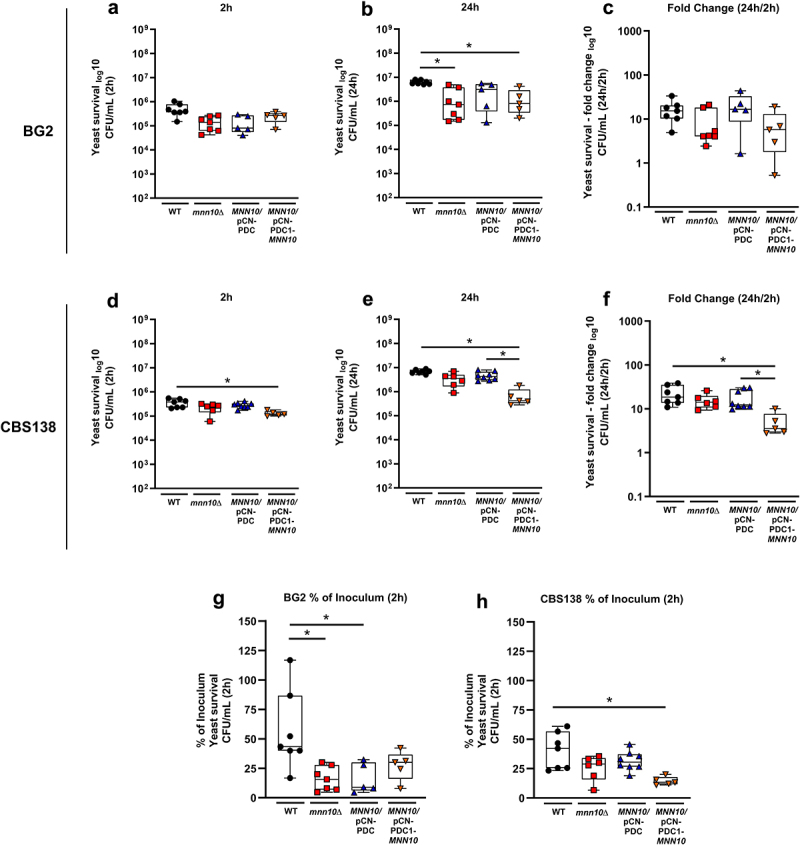
Deletion and overexpression of *MNN10* in both BG2 and CBS138 *N. glabratus* backgrounds show a tendency for reduced survival of yeast cells following BMDM challenge. BMDMs were challenged in technical duplicate at an MOI of 3:1 *N. glabratus* cells to macrophages. Internalized yeast cells were determined at 2 (a, d) and 24 (b, e) hours post-challenge. Data represents the CFU/mL of live yeast recovered by plating at 2 and 24 hours following BMDM interaction. CFU Fold change was determined for yeast recovery at 2 and 24 hours (c, f). Percentage (%) of yeast recovered at 2-hour time-point in relation to the initial inoculum for BG2 (g). Percentage (%) of yeast recovered at 2-hour time-point in relation to the initial inoculum for CBS138 (H). Box and whiskers plot represent minimum to maximum values, showing all points of the dataset. Width distribution of points proportionate to the number of points at that Y value. The whiskers go down to the smallest value and up to the largest; the line in the middle of the box is plotted at the median. *n* = 5–8 biological replicates per group for macrophage yeast survival, mean of technical replicates. *p ≤0.05 between indicated groups. Statistical analyses were done by Kruskal-Wallis with Dunn’s multiple comparisons test.

Thus far, our results indicate that *MNN10* plays an important role in maintaining yeast cell wall architecture (Figures 1 and 2) and limiting host cell activation (Figure 3 and 4, Table 1). Despite the observed reduction in cell damage within the *ex vivo* OECs setting, we wondered whether deletion of *MNN10* would impact *N. glabratus* interaction with immune cells. Additionally, we wanted to explore the implications of altered cell wall architecture in the context of pathogenicity within a whole organism. Considering that the successful interaction of *N. glabratus* with macrophages relies on both intracellular survival and the effectiveness of recognition and phagocytosis, we hypothesized that the variations in cell wall carbohydrate exposure between wild-type and *mnn10*Δ strains would affect macrophage recognition and fungal clearance/killing. To investigate this hypothesis, we subjected bone marrow-derived macrophages (BMDMs) to challenge with *N. glabratus* yeast cells – wild-type, null mutant (*mnn10*Δ), wild-type expressing an extra copy of *MNN10* (*MNN10*/pCN-PDC1-*MNN10*), and wild-type with an empty vector plasmid (*MNN10*/pCN-PDC1). Subsequently, internalized yeasts cells were assessed at 2- and 24-hours post-infection (Figure 5).

Despite increased exposure of all three cell wall polysaccharides, significantly fewer BG2 *mnn10*Δ yeast cells were recovered compared to their respective wild-type control after 2 hours post challenge with BMDMs (Figures 5(a–g)). Interestingly, the BG2 *MNN10*/pCN-PDC1 and *MNN10*/pCN-PDC1-*MNN10* strains were recovered at similar levels as the *mnn10*Δ strain, suggesting that all BG2 strains carrying the pCN-PDC1 plasmid were phagocytosed to a lesser extent compared to the wild-type control (Figures 5(a–g)). Similarly, at 24 hours post-challenge. the BG2 *mnn10*Δ, *MNN10*/pCN-PDC1, and *MNN10*/pCN-PDC1-*MNN10* strains had lower recoverable cells versus the wild-type control (Figure 5(b)). We calculated the fold change in the number of recovered BG2-background yeast cells at 24 hours versus 2 hours post-challenge to investigate intracellular yeast replication. This fold change was reduced for *mnn10*Δ, wild-type plus empty vector and wild-type plus *MNN10* strains versus wild-type only, although these differences were not statistically significant (*p* = 0.9056 for WT vs. *mnn10*Δ; *p* > 0.99 for WT vs. *MNN10*/pCN-PDC1; *p* = 0.7739 for WT vs. *MNN10*/pCN-PDC1-*MNN10*) (Figure 5(c)). These findings suggest that deletion of *MNN10* in the BG2 background leads to reduced early recognition and 2-hour uptake by macrophages, as well as reduced survival and/or replication within phagocytes. However, we cannot draw conclusions about how increased *MNN10* gene dosage affects interaction with BMDMs due to the altered interactions in the empty vector strain.

We observed similar trends in macrophage interactions in the CBS138 background. That is, *MNN10* deletion led to reduced 2-hour uptake by macrophages (Figures 5(d,h)), as well as reduced replication/survival within phagocytes (Figure 5(e)). The fold change between 24 h/2 h showed decreases in CFU for CBS138 *mnn10*Δ yeast cells compared to their wild-type control, although this was not statistically significant (*p* > 0.99 for WT vs. *mnn10*Δ) (Figure 5(f)). Additionally, similar to BG2, the constitutive expression of *MNN10* also led to reduced 2-hour uptake and replication within BMDMs in contrast to the CBS138 wild-type control (Figure 5(d–f)). In contrast to BG2, we did not observe reduced 2-hour uptake and 24-hour recovery for the empty vector strain compared to the wild-type (Figure 5(d–f)). While both strains carrying an extra copy of *MNN10* (*MNN10*/pCN-PDC1-*MNN10*) had a lower fold change in yeast recovery between 24 h/2 h compared to controls, this was only statistically significant for CBS138 strains (*p* = 0.7703 for BG2 *MNN10*/pCN-PDC1 vs. *MNN10*/pCN-PDC1-*MNN10*; *p* = 0.0468 for CBS138 *MNN10*/pCN-PDC1 vs. *MNN10*/pCN-PDC1-*MNN10* on fold change values) (Figures 5(b,c,e,f)).

Overall, our results suggest that *MNN10* is important for mediating *N. glabratus* recognition and survival/replication within macrophages *in vitro*, possibly by controlling exposure of cell wall carbohydrates. However, an extra copy of the gene seems to negatively affect both yeast cell uptake and survival/replication within macrophages without affecting cell wall carbohydrate exposure (Figure 5).

#### Infection with MNN10Δ cells leads to higher mortality and faster melanization kinetics than wild-type cells in *Galleria mellonella* larvae

Finally, in order to fully assess the role of *MNN10* in *N. glabratus* pathogenesis, we investigated virulence in the *G. mellonella* systemic infection model. For this, larvae were infected with yeast cells, and their survival and melanization were assessed for a period of 144 hours post-infection.

**Figure 6.**
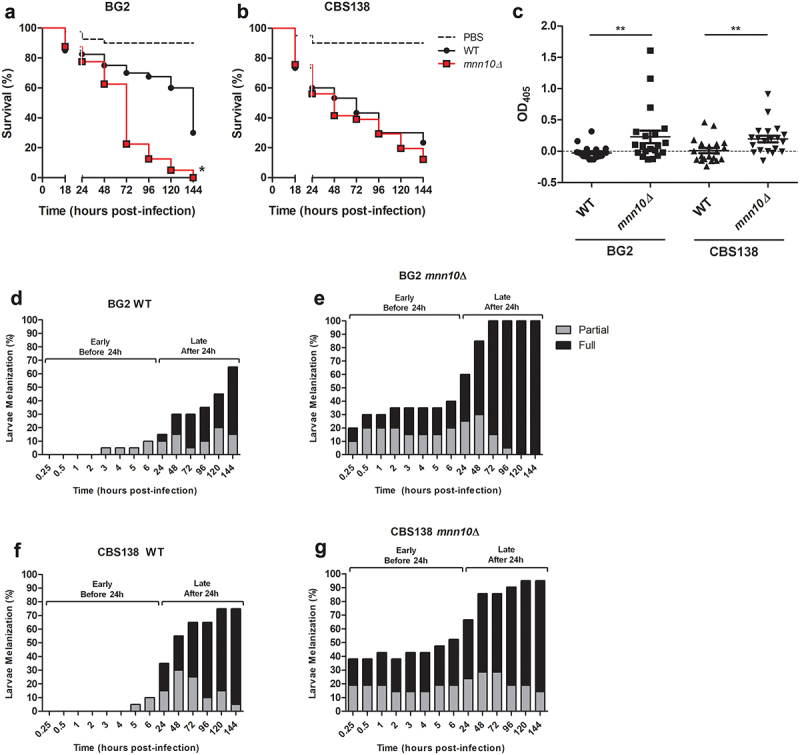
*G. mellonella* larvae infected with *mnn10*Δ cells rapidly melanize and survive at lower rates compared to WT-infected larvae. *G. mellonella* were injected with 5 × 10^6^ BG2 (a) or CBS138 (b) yeast cells grown overnight. Survival was monitored for up to 144 hours post-infection. (c) Hemolymph was collected from *G. mellonella* larvae 2 hours after infection (*n* = 20). Absorbance was measured at 405 nm and is presented normalized to PBS-injected control larvae (*n* = 8). (d-g) melanization of *G. mellonella* larvae was scored as partially or fully melanized [32]. *n* = 30–41 total larvae per group for survival and melanization of *G. mellonella*. PBS (*n* = 40) uninfected control group. WT, wild-type strain. *mnn10*Δ, null mutant strain. *p ≤0.05, **p ≤0.01 between WT and *mnn10*Δ strains. Statistical analysis was performed using Kaplan–Meier test (a-b) and Mann−Whitney U test (c).

BG2 *mnn10*Δ cells were significantly more virulent compared to their wild-type control (Figure 6(a)) and killed the majority of larvae within 72 hours post-infection. CBS138 *mnn10*Δ showed a trend of increased virulence with slightly faster larval death than wild-type cells, but this was not statistically significant (Figure 6(b)).

Strikingly, *G. mellonella* larvae infected with *mnn10*Δ yeast cells from either strain background melanized rapidly (as early as 15 minutes post-infection), whereas the melanization process only started after 3 (BG2) or 5 (CBS138) hours post-infection for larvae challenged with wild-type cells (Figures 6(d–g)). Consistent with this observation, haemolymph collected from *mnn10*Δ-infected larvae 2 hours post-infection had significantly higher absorbance at 405 nm than wild-type-infected larvae (Figure 6(c); *p* ≥ 0.01), suggesting *mnn10*Δ cells enhanced early melanin production. Further, at the end of the 144-hour assessment, 90–100% of the larvae were fully melanized for infections with both BG2 and CBS138 *mnn10*Δ cells (Figures 6(e,g)). Comparatively, approximately 65–75% of larvae infected with wild-type strains were considered fully melanized (Figures 6(d,f)).

These results indicate that *MNN10* deletion results in cell wall or other physiological changes that induce faster melanization of *G. mellonella* larvae and enhance *N. glabratus* virulence in both BG2 and CBS138 backgrounds.

## Discussion

The fungal cell wall is instrumental in modulating host–pathogen interactions. Each of the major cell wall carbohydrates (i.e. chitin, β-glucan, and mannan) can affect host responses through interaction with diverse pattern recognition receptors (PRRs) [43]. Therefore, it is vital to interrogate how diverse cell wall enzymes contribute to the complexity of this structure and ultimately affect disease processes in order to identify potential new avenues for diagnostics or clinical interventions.

One of the prevailing paradigms in fungal-host interactions is that the outer mannan layer shields inner wall components from immune recognition that could induce fungal killing [6]. Much of the research that supports this paradigm was conducted in extracellular fungal pathogens or focused on systemic infection models [2,3,6,7]. In contrast, perturbing *N*-mannan backbone synthesis in *N. glabratus* by deleting *ANP1* or *MNN2* resulted in hypervirulence in systemic infection models [8]. However, the deletion of the MPII complex enzyme, *MNN11*, did not result in yeast hypervirulence [8]. *MNN10* is another MPII complex enzyme involved in extending the mannan backbone in *S. cerevisiae* and has sequence similarity to *MNN11*, but little is known about its role in disease. Thus, our aim was to determine how *MNN10* contributes to *N. glabratus* host recognition and infection in mucosal and systemic disease models.

Our data show that *MNN10* plays a significant role in maintaining *N. glabratus* cell wall architecture and reduces host detectable β-1,3-glucan in the inner cell wall, and thus contributes to context-dependent roles in host immune evasion and fungal pathogenesis. Our argument is based on the following evidence: 1) TEM showing the thinner inner and outer layers of the *mnn10*Δ cell wall compared to wild-type cells, 2) greatly increased detection of β-1,3-glucan, mannan, and chitin in *mnn10Δ* cells by flow cytometry compared to wild-type cells, 3) enhanced phosphorylation of the OEC β-1,3-glucan receptor, EphA2, after challenge with *mnn10*Δ cells, 4) a significant increase in GM-CSF elicitation by BG2 *mnn10*Δ cells and a trend towards decreased LDH release following OEC challenge with both *mnn10*Δ strain backgrounds, 5) a trend towards reduced *mnn10*Δ survival during BMDM challenge, and 6) rapid *G. mellonella* melanization and death after infection with *mnn10*Δ cells.

One of our unexpected findings was that *MNN10* deletion enhanced mannan exposure despite TEM showing a decrease in outer cell wall thickness. Known covalently bound mannoproteins in the *N. glabratus* cell wall include several adhesins and other prevalent proteins, such as Cwp1.1, Cwp1.2, Tir1, and Pir [44]. Cwp1.1 and Cwp1.2 are believed to compose the bulk of the mannoprotein architecture in the wall [44]. Mannoproteins can be decorated with O- and N-mannan, and *MNN10* is believed to function similarly to its *S. cerevisiae* homolog in lengthening the N-mannan fibril. Compensatory O-mannan production could be one potential mechanism that might alter mannan exposure and host interactions in our mutant strains. However, we used concanavalin A to detect mannan, which is a commonly used lectin specific for trimannoside complexes typically associated with high N-mannan structures in fungal cell walls [45,46]. This lectin specificity suggests that the explanation behind enhanced mannan exposure in the mutant is either a compensatory increase in short N-mannan branches, an increase in terminal N-mannan residues, altered permeability that improves N-mannan access, or a combination of these mechanisms. Likewise, high β-glucan and chitin exposure in the *mnn10*Δ may be due to compensatory increases in each carbohydrate, though this would be inconsistent with the reduced inner wall thickness observed by TEM. Rather, our flow cytometry and TEM results are more consistent with the shield model, i.e. that *mnn10*Δ leads to reduced N-mannan extension, and this shorter outer cell wall layer improves the accessibility of β-glucan and chitin to our lectin probes.

Our investigation also revealed important species- and strain-specific differences in fungal-host interactions. *C. albicans* and *N. glabratus* are mucosal commensals and one of the major causes of oral candidiasis. As expected, *C. albicans* induced higher levels of G-CSF, IL-6, IL-1α, and IL-1β than *N. glabratus* cells in an *in vitro* OEC infection model. Surprisingly, both wild-type *N. glabratus* strains elicited higher levels of the pro-inflammatory cytokine GM-CSF from OECs than *C. albicans*. Further, BG2 *mnn10*Δ induced significantly higher GM-CSF than BG2 wild-type cells. We also did not expect CBS138 to cause the most OEC damage of all strains tested. Previous studies have shown that *C. albicans* causes more OEC damage or LDH release than multiple *N. glabratus* isolates, though one study also found that a *N. glabratus* clinical isolate induced high levels of GM-CSF [47,48,49]. These differences in host damage may be because our study challenged TR146 cells whereas other studies used combinations of OKF6/TERT-2 cells or reconstituted epithelium with TR146 and squamous keratinocytes. Overall, our cytokine and LDH data suggest that *N. glabratus MNN10* modulates oral mucosal immune evasion, potentially by controlling yeast cell wall polysaccharide exposure and limiting β-1,3-glucan recognition by host cells.

**Figure 7.**
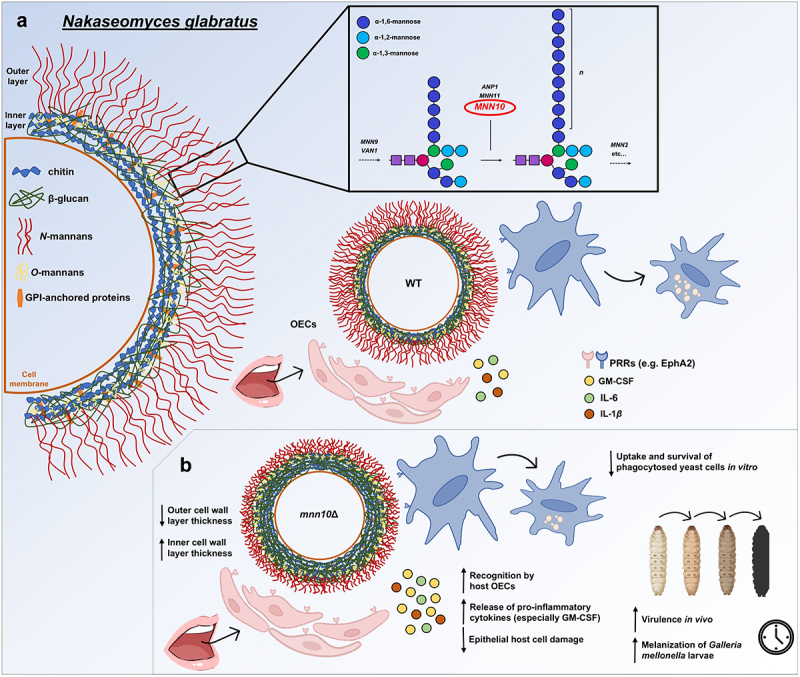
Mannan is a context-dependent shield that modifies virulence in *N. glabratus*. (a) Mannan elongation is important for normal cell wall organization and host interactions. Mannan fibril elongation is orchestrated by a series of enzymes that include Anp1, Mnn11, and Mnn10. (b) *MNN10* deletion alters cell wall architecture and increases carbohydrate exposure. The enhanced β-1,3-glucan exposure on *mnn10*Δ cells triggers greater host recognition and cytokine release. These altered host responses are protective in certain models, such as oral epithelial cell or macrophage challenge, but appear to be deleterious in systemic infection.

Strikingly, *G. mellonella* infection with *mnn10*Δ cells led to a lower larval survival rate compared to wild-type cells (Figure 6), though the survival difference was only significant in the BG2 background. Mutants in both backgrounds induced rapid larval melanization, suggesting that *G. mellonella* may be mounting an inappropriate heightened inflammatory response to infection with *mnn10*Δ cells. These data, while in a different host model, are consistent with previous work showing that *ANP1* and *MNN2* deletion mutants are hypervirulent during murine systemic infection. Thus far, *ANP1*, MNN2, and *MNN11* mutants have not been tested in multiple strain backgrounds. While the cell wall alterations we observed for *mnn10*Δ cells were similar in reference strain backgrounds, the strength of our *MNN10* virulence phenotypes was strain-dependent, therefore it is important to test the role of cell wall modifying enzymes in multiple strain backgrounds to generate robust conclusions about cell wall remodelling and virulence. Ultimately, most of our data support the prevailing paradigm that the mannan outer layer shields inner cell wall components from initiating host recognition and fungal killing (Figure 7). However, the different outcomes we observed in each host model also indicate that the context or specific niche for host-fungal interactions affects whether *N. glabratus* cells conform to this paradigm. While *MNN10* deletion led to better host cell outcomes in *in vitro* oral and BMDM infection models compared to wild-type infections, these mutants induced rapid death in *G. mellonella* larvae during systemic infection. Thus, our data taken alongside previous evidence suggest that mannan backbone extension plays a vital role in mediating *N. glabratus* host interactions during infection and highlights how cell wall-targeted interventions may differentially impact host outcomes in mucosal and systemic disease.

## Supplementary Material

Supplemental Material

Supplemental Material

Supplemental Material

Supplemental Material

ARRIVE_Checklist.pdf

## Data Availability

The data supporting the findings in this study are available within the paper and accompanying supplemental material. Raw data supporting all figures are available at FigShare (https://doi.org/10.6084/m9.figshare.26150014).
