# Supplementary material for: Mannan is a context-dependent shield that modifies virulence in Nakaseomyces glabratus
Source: Virulence. 2025 Apr 15;16(1):2491650. doi: 10.1080/21505594.2025.2491650 (PMC12001547; doi:10.1080/21505594.2025.2491650)
Supplement: Supplemental Material [file KVIR_A_2491650_SM6929.docx]

**Table S2. Primers used in this study.** For deletion primers, uppercase letters represent homologous nucleotides upstream of the *MNN10* gene sequence; lowercase letters represent specific sites for CLOX cassette amplification. For ORF primers, *MNN10* forward primers with SpeI cut site; reverse primers with XhoI cut site.

| **Name** | **Sequence 5’-3’** |
| --- | --- |
| **Deletion Primers** | |
| ***MNN10* Forward** | ccgtatttagagggggtggactggttcaaactgggttggtagcattgttaagtgtagcacccgagtatt  actctgacaagaaaagtttctactaagagccACGGCCAGTGAATTGTAATA |
| ***MNN10* Reverse** | tcctgacaaatattcttcctggtccaagactgctcatgaaaccgcagattttataataag  atcactataccgattcaaattgaaactacctgagatgcatTCGGAATTAACCCTCACTAA |
| ***MNN10* Confirmation Forward** | TAGGAAGACTTCGATGCC |
| ***MNN10* Confirmation Reverse** | GGATACTTTCTCAATGGC |
| **ORF Primers for pCN-PDC1 Cloning and Deletion/Ectopic Expression Confirmation Post-Transformation** | |
| ***MNN10* Forward** | aacatctagaactagtATGTCAAGGAGGGCCTCGC |
| ***MNN10* Reverse** | gggttgtgttctcgagCTAAATAAAGAAGAATAGC |
