# Supplementary material for: Mannan is a context-dependent shield that modifies virulence in Nakaseomyces glabratus
Source: Virulence. 2025 Apr 15;16(1):2491650. doi: 10.1080/21505594.2025.2491650 (PMC12001547; doi:10.1080/21505594.2025.2491650)
Supplement: Supplemental Material [file KVIR_A_2491650_SM6903.docx]

**Table S3. Freeze substitution protocol.** Standard freeze sub cocktail, 1% osmium tetroxide (OsO_4_) in acetone. Additional contrast, 0.2% Glutaraldehyde, 0.1% Uranyl Acetate in acetone, 2% Methylcyclohexane, 1% H_2_O in acetone.

| **Step** | **Start Temp. (^o^C)** | **End Temp. (^o^C)** | **Time (Hours:Minutes)** | **Reagent** |
| --- | --- | --- | --- | --- |
| **1** | -95 | -90 | 30 | Freeze sub cocktail |
| **2** | -90 | -90 | 10:00 | Freeze sub cocktail |
| **3** | -90 | -30 | 08:00 | Freeze sub cocktail |
| **4** | -30 | -10 | 01:00 | Acetone |
| **5** | -10 | 4 | 01:00 | Acetone |
| **6** | 4 | 20 | 01:00 | Acetone |
