# Supplementary material for: Mannan is a context-dependent shield that modifies virulence in Nakaseomyces glabratus
Source: Virulence. 2025 Apr 15;16(1):2491650. doi: 10.1080/21505594.2025.2491650 (PMC12001547; doi:10.1080/21505594.2025.2491650)
Supplement: Supplemental Material [file KVIR_A_2491650_SM6901.docx]

**Table S1. List of all strains used in the study.**

| **Strain** | **Genotype** | **Parent** | **Source** |
| --- | --- | --- | --- |
| **BG2** | *N. glabratus* Wild-Type | - | (Cormack & Falkow, 1999) |
| **CBS138 (ATCC2001)** | *N. glabratus* Wild-Type | - | (Dujon et al., 2004; Koszul et al., 2003) |
| **SC5314** | *C. albicans* Wild-Type |  | (Gillum et al., 1984) |
| **BG2 *mnn10*Δ** | *mnn10Δ*::loxP | BG2 WT | This study |
| **BG2 *MNN10*/pCN-PDC1-*MNN10*** | *MNN10*/pCN-PDC1-*MNN10* | BG2 WT | This study |
| **BG2 *mnn10*Δ/pCN-PDC1-*MNN10*** | *mnn10*Δ::loxP/pCN-PDC1-*MNN10* | BG2 *mnn10*Δ | This study |
| **BG2 *MNN10*/pCN-PDC1** | *MNN10*/pCN-PDC1 | BG2 WT | This study |
| **CBS138 *mnn10*Δ** | *mnn10Δ*::loxP | CBS138 WT | This study |
| **CBS138 *MNN10*/pCN-PDC1-*MNN10*** | *MNN10*/pCN-PDC1-*MNN10* | CBS138 WT | This study |
| **CBS138 *mnn10*Δ/pCN-PDC1-*MNN10*** | *mnn10*Δ::loxP/pCN-PDC1-*MNN10* | CBS138 *mnn10*Δ | This study |
| **CBS138 *MNN10*/pCN-PDC1** | *MNN10*/pCN-PDC1 | CBS138 WT | This study |
