# Supplementary figures and images for: Mannan is a context-dependent shield that modifies virulence in Nakaseomyces glabratus
Source: Virulence. 2025 Apr 15;16(1):2491650. doi: 10.1080/21505594.2025.2491650 (PMC12001547; doi:10.1080/21505594.2025.2491650)

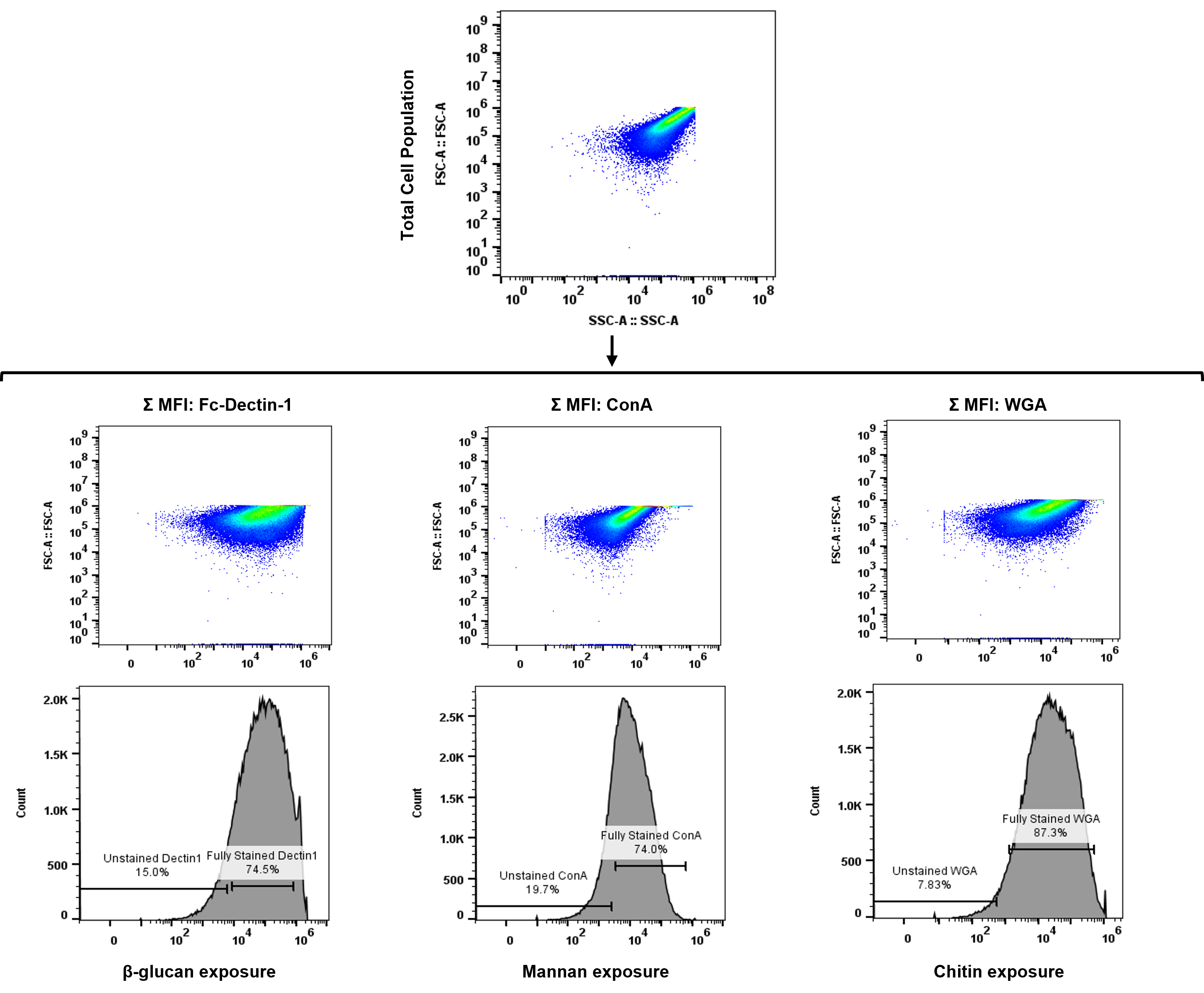

Supplement: Supplemental Material [file KVIR_A_2491650_SM6900.tiff]
